# Changes in the biochemical and immunological components of serum and colostrum of overweight and obese mothers

**DOI:** 10.1186/s12884-015-0574-4

**Published:** 2015-08-12

**Authors:** Mahmi Fujimori, Eduardo L. França, Vanessa Fiorin, Tassiane C. Morais, Adenilda C. Honorio-França, Luiz C. de Abreu

**Affiliations:** Department of Maternal and Child Health, School of Public Health, University of São Paulo, São Paulo, SP Brazil; Institute of Biological and Health Science, Federal University of Mato Grosso, Barra do Garças, MT Brazil; Laboratory of Scientific Writing, Department of Morphology and Physiology, School of Medicine of ABC, Santo André, SP Brazil

**Keywords:** Colostrum, Obesity, Antibody, Complement protein, Fat

## Abstract

**Background:**

Obesity in pregnancy is associated with systemic inflammation, immunological changes and adverse maternal-fetal outcomes. Information on the association between maternal obesity and breast milk composition is scarce. This study describes changes and relationships between biochemical and immunological parameters of colostrum and serum of overweight and obese women.

**Methods:**

Colostrum and blood samples were collected from 25 normal weight, 24 overweight and 19 obese women for determination of glucose, total protein, triglycerides, cholesterol, immunoglobulins, complement proteins (C3 and C4), fat and calorie content and C-reactive protein (CRP).

**Results:**

Glucose was higher in colostrum of obese women (*p* = .002). In normal weight and obese women, total protein content was higher in colostrum than in serum (*p* = .001). Serum triglycerides (*p* = .008) and cholesterol (*p* = .010) concentrations were significantly higher in overweight and obese women than in their normal weight counterparts, but in colostrum their concentrations were similar across the three groups. Secretory IgA (sIgA) in colostrum and IgA in serum concentrations were significantly higher (*p* = .001) in overweight and obese mothers, whereas IgG and IgM concentrations did not vary among the groups (*p* = .825). Serum C3 (*p* = .001) and C4 (*p* = .040) concentrations were higher in obese women. No differences in colostrum complement proteins were detected among the groups. Calorie content (*p* = .003) and fat (*p* = .005) concentrations in colostrum and serum CRP (*p* = .002) were higher in obese women.

**Conclusions:**

The results corroborate the hypothesis that colostrum of overweight and obese women undergoes biochemical and immunological changes that affect its composition, namely increasing glucose concentrations, calorie content, fat and sIgA concentrations.

## Background

Breastfeeding promotion is one of the main strategies for reducing child mortality worldwide [1]. Breast milk contains a balanced content of macro and micronutrients that are essential for infant growth and development, and several immunological components that provide protection to newborns [2–4]. Antibodies, complement proteins, hormones, immunocompetent cells [3, 5–9] cytokines [10–12] and other milk components appear to play a role in the modulation and development of the immune system and inflammatory responses of newborns.

In recent decades, due to changes in lifestyle the incidence of overweight and obesity are increasing among breastfeeding mothers. Obesity and overweight are common metabolic disorders and their growing frequencies worldwide are a major public health concern [13]. Obesity has been shown to increase the expression and secretion of proinflammatory cytokines such as tumor necrosis alpha factor (TNF-α) and interleukin 6 (IL-6) and raises plasma C-reactive protein (CRP) concentrations, leading to the chronic low-grade inflammation that characterizes the disease [14] and appears to play a central role in the development of a variety of metabolic disorders and hormonal dysfunctions [15–17]. The fact that obesity affects components of the cellular and humoral immune response may result in a state of immunodeficiency [18]. Epidemiological and clinical findings corroborate this, showing a higher incidence and severity of infectious diseases in the obese [19].

The risks associated with obesity become even more relevant in women of reproductive age [20, 21] because pregnancy is another condition that affects immune response. Moreover, excess weight during pregnancy contributes to increasing perinatal morbidity and mortality, posing risks of long-term consequences for mother and child [22].

Other studies show that chronic inflammation caused by obesity may be related to an exaggerated inflammatory response in the placenta of pregnant women, with an accumulation of macrophages and pro-inflammatory mediators [23]. However, it is unclear whether the effects of obesity in pregnancy are accompanied by changes in colostrum composition in the post partum period.

Changes in maternal metabolism and immunological response may affect the developing immune system of newborns because of the intense interplay between mother and child during pregnancy and breastfeeding. To clarify this issue, the present study investigated the association between maternal weight and immunological, biochemical and nutritional parameters of colostrum.

## Methods

This cross-sectional study evaluated 68 mothers (18–36 years of age), divided into three groups according to their prepregnancy body mass index (BMI): normal weight (*n* = 25); overweight (*n* = 24) and obese (*n* = 19). Group definition was based on the World Health Organization [24] criterion, considering normal weight for BMI 18.5–24.9, overweight for BMI 25–29.9, and obesity for BMI of 30 or more. Participants were recruited from the Pregnancy and Obstetric Service of the Maria José dos Santos Stein Hospital, managed by the School of Medicine of ABC, Santo André, SP, Brazil. The volunteers signed an informed consent form before entering the study, which was approved by the local ethics committee of the Faculty of Public Health of the University of São Paulo (Protocol Number CAAE: 05269612.7.0000.5421).

A number of variables were controlled in both groups. These patients were characterized by the age, gestational age at delivery, smoking status, hypertension, body mass indexes before pregnancy, diabetes prior to pregnancy and gestational diabetes.

### Inclusion and exclusion criteria

The inclusion criteria were as follows: (a) women with breasts without nipple fissures or mastitis; (b) who were exclusively breastfeeding their babies; and (c) signed a Consent Form. Women with multiple pregnancies, fetal malformations and deliveries before the 37th week of gestation were excluded.

### Colostrum sampling

About 48–72 h post-partum, 10 mL colostrum was collected from the volunteers. Supernatant was obtained by colostrum centrifugation at 160 × g for 10 min at 4 °C. The upper fat layer was discarded, and the aqueous supernatant was stored at −80 °C for later biochemical and immunological analyses.

### Blood sampling

Samples of 10 mL of blood were collected prior to the beginning of labor from each mother in tubes without anticoagulant and the blood samples were centrifuged at 160 × g for 15 min, until serum separation. Serum samples were stored individually at −80 °C for further glucose, enzyme and protein determination.

### Glucose determination

Glucose concentrations of colostrum supernatant and maternal serum were determined by the enzymatic system [25]. Samples of 20 μL colostrum, serum and standard of 100 mg/dL (BioTécnica®, Ref 10.008.00, Brazil) were placed in 2.0 mL phosphate buffer solution (0.05 M, pH7.45, with aminoantipyrine 0.03 mM, 15 mM sodium p-hydroxybenzoate, 12 kU/L glucose oxidase and 0.8 kU/L peroxidase). The suspensions were mixed and incubated for 5 min at 37 °C. The reactions were read on a spectrophotometer at 510 nm.

### Total protein determination

Total protein of colostrum supernatant and maternal serum was determined by the Biuret colorimetric method [25]. Samples of 20 μL of colostrum, serum and standard of 4 g/dL (BioTécnica®, Ref 10.009.00, Brazil) were placed in 1.0 mL Biuret reagent (ions of copper in alkaline medium). The suspensions were mixed and incubated for 10 min at 37 °C. The reactions were read on a spectrophotometer at 545 nm.

### Cholesterol determination

Cholesterol concentrations of colostrum supernatant and maternal serum were determined by enzymatic colorimetric method [26]. Samples of 10 μL colostrum/serum and standard of 200 mg/dL BioTécnica®, Ref 10.004.00, Brazil), were placed in 1 mL of buffer solution (100 mmol/L, pH 7.0; Sodium cholate 8 mmol/L; cholesterol esterase 750 U/L; Cholesterol oxidase/200 U/L; Peroxidase > 2000 U/L; 4-aminoantipyrine 0.6 mmol; phenol 20 mmol /L; Sodium azide 0.05 % v/v). The suspensions were mixed and incubated for 10 min at 37 °C. The reactions were read on a spectrophotometer at 505 nm.

### Triglycerides determination

Triglycerides concentrations in colostrum supernatant and maternal serum were determined by the enzymatic colorimetric method [26]. Samples of 10 μL of colostrum/serum, standard of 200 mg/dL (BioTécnica®, Ref 10.010.00, Brazil), were placed in 1.0 mL of buffer solution (50 mmol/L pH 7.2 Glicerol kinase/1000 U/L; Peroxidase/1000 U/L; Lipoprotein lipase/2000 U/Ll; Glycerol-3-phosphate oxidase/5000 U/L; 4-chlorophenol 2.7 mmol/L; 4-aminoantipyrine 0.3 mmol/L; ATP - adenosine triphosphate 2.0 mmol/L; Sodium azide 0.01 % v/v). The suspensions were mixed and incubated for 10 min at 37 °C. The reactions were read on a spectrophotometer at 505 nm.

### Immunoglobulin, C3 and C4 complement determination

The immunoglobulin (Ig), complement protein (C) 3 and 4 concentrations in colostrum and serum were determined by turbidimetric method [11].

For sIgA in colostrum and IgA in serum determinations, the samples were diluted at 1:5 (v/v) with saline solution (9 g/L), for IgM at 1:11 (v/v) and for IgG at 1:15 (v/v), and the antibody concentrations were determined using IgA (Bioclin®, Brazil, Ref K061), IgM (Bioclin®, Brazil, Ref K063) and IgG antiserum (Bioclin®, Brazil, Ref K062) diluted at 1:12 (v/v). A calibration curve obtained by the Multical (Bioclin®, Brazil, Ref K064) calibrator was used to determine the standard curve for each immunoglobulin. Samples of colostrum, serum, standards, positive and negative control sera were placed in 500 μL of buffer solution (sodium chloride 0.15 moL/L, Tris 50 mmol/L, 6.0000 PEG 50 g/L, sodium azide 15.38 nmol/L). The suspensions were mixed and incubated at 37 °C for 10 min. The reactions were read on a spectrophotometer at 340 nm.

For C3 and C4 concentration determination the samples (colostrum and serum) were diluted at 1:12 (v/v) with saline solution (9 g/L), and the C3 and C4 concentrations in sample supernatants were determined using C3 and C4 antiserum (Bioclin®, Brazil) diluted at 1:12 (v/v). A calibration curve obtained by the Multical (Bioclin®, Brazil), Ref K064) calibrator was used to determine the standard curve. Ten microliter samples of colostrum, serum, standards, positive and negative control sera were placed in 500 μL of buffer solution (sodium chloride 0:15 mol/L, Tris 50 mmol/L, 6.0000 PEG 50 g/L, sodium azide 15:38 nmol/L). The suspensions were mixed and incubated at 37 °C for 15 min. The reactions were read on a spectrophotometer at 340 nm.

### Creamatocrit analysis

Colostrum samples were water-bath-heated at 40 °C for 15 min and subjected to vortex mixing. Capillary tubes (2 μL) were filled to approximately three quarters with the samples, sealed with sealing wax and centrifuged for 15 min. Centrifugation separated the samples into cream and serum [2]. The cream column and the total column were measured, and fat and Kcal content calculated using the following formulae:

$$ \mathrm{Fat}\ \mathrm{content}\kern0.5em =\kern0.5em \%\mathrm{cream}\kern0.5em -\kern0.5em 0.59/1.46,\ \mathrm{where}\ \mathrm{the}\ \%\ \mathrm{cream}\kern0.5em =\kern0.5em \mathrm{cream}\ \mathrm{column}\ \left(\mathrm{mm}\right)\kern0.5em \times \kern0.5em 100\ /\mathrm{total}\ \mathrm{column}\ \left(\mathrm{mm}\right); $$

$$ \mathrm{Kcal}/\mathrm{L}\kern0.5em =\kern0.5em \left(68.8\times \%\mathrm{cream}\right)\kern0.5em +\kern0.5em 290 $$

### C-Reactive Protein assay

C-Reactive Protein (CRP) concentrations in human colostrum and serum were measured using the PCR Turbilatex Kit (BioTécnica®, Brazil, Catalog 20.015.00) by turbidimetric method [26]. Samples of 5 μL of colostrum, serum and standard were placed in 1000 μL of solution (phosphate Buffer 40 mmol, sodium azide 0.95 g/L, suspension of latex particles sensitized with goat IgG anti Human C-reactive protein). The suspensions were mixed and placed at 37 °C and the reactions were measured immediately and at 120 s. The reactions were read on a spectrophotometer at 540 nm.

### Statistical analysis

Two-way Analysis of variance (ANOVA) with calculation of F statistic and Tukey’s multiple comparison test were used to evaluate glucose, total protein, cholesterol, triglycerides, antibody concentration, complement protein, CRP, calories and fat considering the BMI status as one factor and the biological materials (colostrum or serum) as the other. Statistical significance was considered when the *p*-value was less than .05.

## Results

Clinical characteristics from all groups are shown in Table 1. Maternal age, gestational age at birth and height before pregnancy were similar among groups. The cesarean birth percentage was higher in overweight and obese groups. Neonates from the groups studied (normal weight, overweight and obese) exhibited similar somatometry at birth (Table 1).

**Table 1:** Clinical characteristics of woman included in the study according to pregestacional BMI group (normal, overweight and obese)

Variables	Normal weight (*n* = 25)	Overweight (*n* = 24)	Obese (*n* = 18)
Mothers			
Age (year)	25.0 (18–37)	24.1 (18–37)	26.8 (21–38)
height before pregnancy (cm)	161.0 (150.0-171.0)	162.8 (150.0-172.0)	160.4 (144.0-189.0)
Weight before pregnancy (kg)	56.2 (43.0-68.5)	71.2 (56.5-82.0)	87.3 (66.0-110.0)
BMI before pregnancy (kg/m^2^)	21.4 (18.4-24.4)	26.6 (25.2-28.6)	34.7 (30.1-47.9)
Cesarean (%)	6/25 (24.0 %)	11/24 (45.8 %)	10/18 (55.5 %)
Gestational age at delivery (week)	39.7 (37.0-41.3)	39.5 (37.6-41.0)	39.5 (37.0-40.7)
Primipara (%)	13/25 (52.0 %)	11/24 (45.8 %)	8/18 (44.4 %)
Diabetes	0/25 (0.0 %)	0/24 (0.0 %)	0/18 (0.0 %)
Gestational diabetes	0/25 (0.0 %)	0/24 (0.0 %)	2/18 (11.0 %)
Hypertension	1/25 (4.0 %)	1/24 (4.0 %)	2/18 (11.0 %)
Smoking status	4/25 (16.0 %)	2/24 (8.0 %)	2/18 (11.0 %)
Infants			
Infant gender (% female)	12/25 (48.8 %)	10/24 (41.6 %)	8/18 (44.4 %)
Weight (kg)	3.6 (2.40-4.20)	3.2 (2.54-3.91)	3.4 (2.54-4.34)
Height (cm)	48.1 (44.0-52.0)	48.2 (44.5-53.0)	49.2 (46–52)

Data for all mothers included are shown as median, minimum and maximum values or number and percentages (%)

We evaluated biochemical (Table 2) and immunological (Table 3) parameters in the blood and colostrum of mothers with different body mass index (BMI).

**Table 2:** Biochemical parameters in colostrum and serum from normal weight, overweight and obese mothers

Parameter		Normal weight	Overweight	Obese	Statistical
Glucose concentrations (mmol/L)	Colostrum	1.9 (0.7–2.9) ^3^	2.6 (2.1–3.1) ^1,3^	3.2 (2.0–4.5) ^1,2,3^	F = 5.94; *p* = .002 (comparing the groups)
	Serum	4.3 (4.2–5.1)	4.4 (3.7–5.4)	4.3 (3.7–5.7)	F = 139.15; *p* = .001 (comparing the colostrum and serum)
Total Protein (g/L)	Colostrum	101.0 (91.0–115.0) ^3^	89.0 (76.0–127.0)	86.0 (61.0–157.0) ^3^	F = 0.25; *p* = .758 (comparing the groups)
	Serum	59.0 (57.0–67.0)	62.5 (58.0–83.0)	66.5 (54.0–76.0)	F = 47.53; *p* = .001 (comparing the colostrum and serum)
Triglyceride concentrations (mmol/L)	Colostrum	5.3 (2.5–6.6) ^3^	5.1 (2.5–6.3) ^3^	4.6 (2.6–8.0) ^3^	F = 10.59; *p* = .008 (comparing the groups)
	Serum	1.6 (0.8–2.2)	1.9 (0.9–3.3)	2.9 (2.0–5.5) ^1^	F = 57.47; *p* = .001 (comparing the colostrum and serum)
Cholesterol concentrations (mmol/L)	Colostrum	4.3 (2.1–6.2)	4.1 (2.7–5.9)	5.2 (2.9–8.2) ^3^	F = 5.05; *p* = .010 (comparing the groups)
	Serum	5.1 (4.7–5.4)	5.4 (4.6–5.9)	6.0 (5.2–12.3) ^1^	F = 11.08; *p* = .002 (comparing the colostrum and serum)
CRP concentrations (mg/L)	Colostrum	4.0 (0.0–8.0)	5.0 (0.0–11.0)	6.0 (0.0–12.0)	F = 12.30; *p* = .002 (comparing the groups)
	Serum	9.0 (5.0–28.0)	16.0 (0.0–26.0) ^3^	85.0 (17.0–201.0) ^1,2,3^	F = 20.71; *p* = .001 (comparing the colostrum and serum)
Fat (%)	Colostrum	3.3 (0.4–6.7)	3.6 (1.2–9.4)	5.6 (2.0–11.9) ^1^	F = 7.27; *p* = .005 (comparing the groups)
Calories (Kcal)	Colostrum	537.9 (396.7–764.3)	543.6 (396.7–944.6)	688.2 (450.3–1111.6) ^1^	F = 6.90; *p* = .003 (comparing the groups)

Data presented as media, minimum and maximum values

^1^Statistically significant differences in relation to the normal weight category, considering the same sample (colostrum or serum)

^2^Statistically significant differences in relation to the overweight category, considering the same sample (colostrum or serum)

^3^Statistically significant differences between colostrum and serum, considering the same group (normal weight, overweight and obese)

**Table 3:** Immunoglobulins and complement protein concentrations in colostrum and serum from normal weight, overweight and obese mothers

Parameter		Normal weight	Overweight	Obese	Statistical
IgA (g/L)	Colostrum	3.3 (2.3-5.5) ^3^	3.8 (2.1-5.0)	5.1 (3.3-9.6) ^1,2,3^	F = 12.44; *p* = .001 (comparing the groups)
	Serum	2.2 (1.6-3.1)	2.7 (2.2-3.5)	3.9 (2.1-4.7) ^1^	F = 21.69; *p* = .002 (comparing the colostrum and serum)
IgM (g/L)	Colostrum	1.3 (0.85-2.2)	1.2 (0.8-2.1)	1.4 (1.0-3.4)	F = 0.19; *p* = .825 (comparing the groups)
	Serum	1.3 (1.1-1.8)	1.4 (0.8-2.4)	1.0 (0.8-2.0)	F = 1.84; *p* = .177 (comparing the colostrum and serum)
IgG (g/L)	Colostrum	0.4 (0.1-0.6) ^3^	0.3 (0.2-0.5) ^3^	0.4 (0.2-0.6) ^3^	F = 0.05; *p* = .947 (comparing the groups)
	Serum	11.3 (9.2-14.2)	9.3 (8.5-16.7)	10.8 (7.6-18.4)	F = 495.8; *p* = .001 (comparing the colostrum and serum)
C3 (mg/dL)	Colostrum	91.7 (50.5-110.2) ^3^	90.3 (44.1-97.9) ^3^	95.7 (41.3-130.9) ^3^	F = 14.98; *p* = .001 (comparing the groups)
	Serum	121.1 (109.5-139.3)	152.4 (90.6-250.0)	249.8 (119.1-312.9) ^1,2^	F = 117.30; *p* = .001 (comparing the colostrum and serum)
C4 (mg/dL)	Colostrum	30.1 (15.6-34.8) ^3^	24.2 (19.6-40.7) ^3^	28.8 (17.7-40.2)	F = 5.91; *p* = .040 (comparing the groups)
	Serum	16.4 (9.9-25.6)	17.2 (7.1-25.8)	22.9 (13.3-37.1) ^1,2^	F = 20.68; *p* = .001 (comparing the colostrum and serum)

Data presented as median, minimum and maximum values (within parentheses)

^1^Statistically significant differences in relation to the normal weight category, considering the same sample (colostrum or blood)

^2^Statistically significant differences in relation to the overweight category, considering the same sample (colostrum or blood)

^3^Statistically significant differences between colostrum and serum, considering the same group (normal weight, overweight and obese)

Colostrum glucose concentrations were higher in obese (*p* = .002) than in overweight and normal weight groups. Serum glucose concentrations were higher (*p* = .001) than colostrum glucose concentrations in overweight mothers (Table 2). Total protein concentrations were similar (*p* = .758) across the groups. In normal weight and obese groups, total protein concentrations were higher in colostrum (*p* = .001) than in maternal serum (Table 2).

Intergroup colostrum triglyceride concentrations were similar, but in serum they were significantly higher (*p* = .008) in the obese group. Irrespective of the BMI, triglyceride concentrations were higher (*p* = .001) in colostrum than in serum (Table 2). No statistical intergroup differences (*p* > 0.05) in cholesterol concentrations were detected in colostrum. However, obese women exhibited higher cholesterol concentrations in serum (*p* = .010) than normal weight individuals. In the obese group, cholesterol concentrations were higher in serum (*p* = .001) than in colostrum (Table 2).

**Fig. 1:**
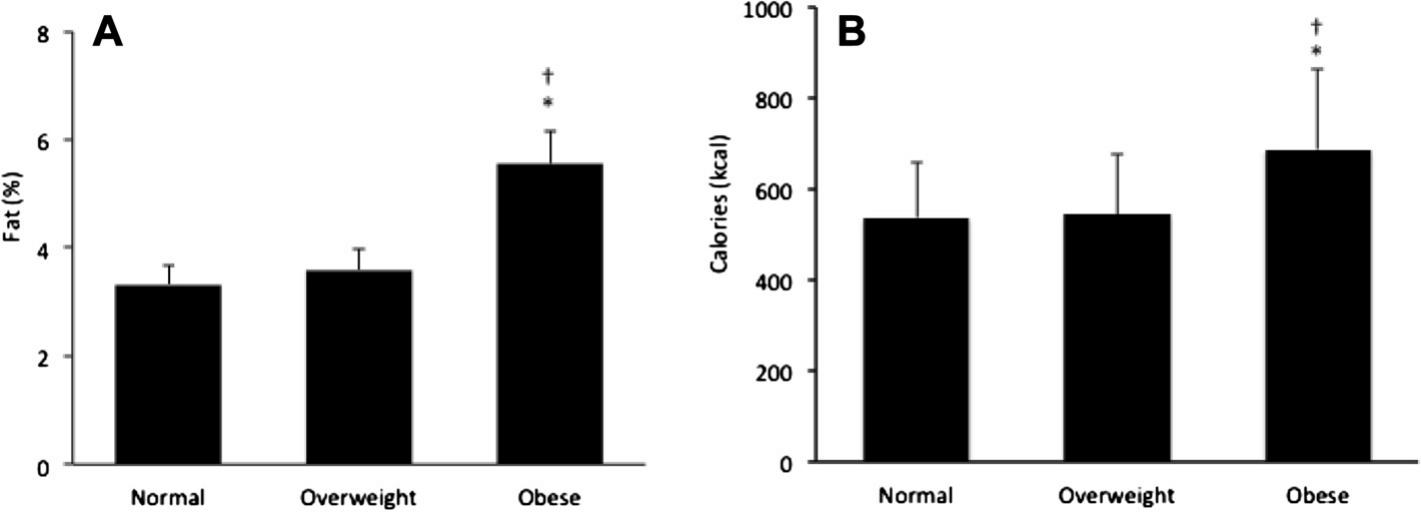
Fat **a** and calories **b** in the colostrum of normal, overweight and obese mothers. Data presented as mean ± standard error (SE). F(A) = 7.27; *p* = .005; F(B) = 6.90; *p* = .003. *Statistically significant differences between normal and obese groups. †Statistically significant differences between overweight and obese groups

**Fig. 2:**
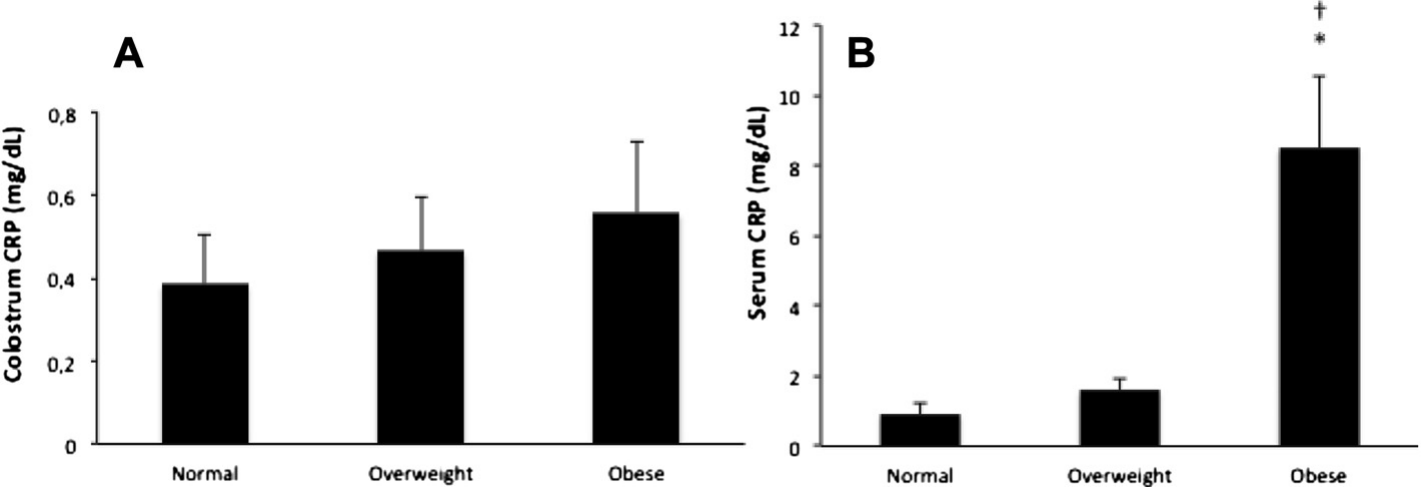
CRP concentrations in the colostrum **a** and serum **b** of normal, overweight and obese mothers. Data presented as mean ± standard error (SE). F = 12.30; *p* = .002 comparing the groups; F = 20.71; *p* = .001 comparing the samples (colostrum and serum). * Statistically significant differences between normal and obese groups. †Statistically significant differences between overweight and obese groups

As shown in Fig. 1 and Table 2, fat (*p* = .005) and calorie (*p* = .003) content of colostrum was higher in the obese group. Colostrum CRP concentrations were similar among the groups, but in serum, they were significantly higher in the obese group (*p* = .002) than in the other groups. The highest concentrations of CRP (*p* = .001) were found in serum (Fig. 2 and Table 2).

The obese group showed significantly higher (*p* = .001) colostrum sIgA and serum IgA concentrations than normal weight women. IgM concentrations did not vary (*p* = .825) among the groups and between serum and colostrum samples (*p* = .177). IgG concentrations were significantly higher in serum (*p* = .001) than in colostrum (Table 3).

In serum, the concentrations of complement proteins C3 (*p* = .001) and C4 (*p* = .040) were significantly higher (*p* = .010) in the obese group than in normal weight and overweight groups, but in colostrum did not differ among the groups. Irrespective of weight status, C3 concentrations were significantly higher in serum (*p* = .001) than in colostrum, and in normal weight and overweight groups, C4 concentrations were significantly higher in serum (*p* = .001) than in colostrum samples (Table 3).

## Discussion

The immune protection that breast milk provides and its nutritional importance has been widely described, and the composition of this secretion is known to undergo inter-and intra-individual variations [27]. These variations in breast milk can be affected by several factors, such as maternal diet, nutritional status, smoking, parity and period of the day [2, 28, 29].

Other studies report that maternal anemia, hypertension and diabetes can change nutritional and immunological features of breast milk [11, 30–32]. The present study shows that although serum cholesterol, triglycerides, CRP, C3 and C4 protein concentrations were higher in obese women, their concentrations in colostrum were similar across the groups. On the other hand, although glucose and IgA concentrations were similar among serum samples of the different groups, in colostrum samples sIgA concentrations were significantly higher in obese mothers. An increase in glucose concentrations in the breast milk of diabetic women are associated with long-term consequences for their children, such as an increase in weight gain and metabolic changes [33, 34]. This suggests that infants born to and breastfed by women with high prepregnancy BMI may be heavier and predisposed to develop obesity and related disorders in adulthood. Other studies should investigate the effects of maternal weight on the glucose content of breast milk and its impact on infant weight gain and metabolism.

The association between maternal obesity and dyslipidemia has been extensively described [35–37], and it likely contributes to vascular diseases including preeclampsia and the development of macrosomia [38]. The present study also found increased of dyslipidemia markers in higher BMI groups in colostrum samples, given that in obese and overweight groups this secretion contained higher fat and calorie content compared to normal weight women. The effects of maternal BMI on the energy content of breast milk are controversial, with studies showing that fat content in the milk of obese women does not differ from that of other weight classes [39], whereas others report lower fat content in milk from overweight mothers [28].

The immaturity of the immune system of newborns makes it susceptible to infections by viruses and bacteria. Accordingly, the transfer of antibodies *via* placenta during uterine life and then *via* colostrum and breast milk after birth is important in reducing this deficiency [30]. In the present study, overweight and obese mothers exhibited higher IgA concentrations in serum and sIgA concentrations in colostrum than normal weight women. Earlier studies report that obesity increases serum IgA concentrations in both sexes [40]. The present study is the first to evaluate the concentrations of antibodies and complement proteins in colostrum of overweight pregnant women. The increase in sIgA concentrations in colostrum might be associated with conditions determined by the metabolic syndrome, including hyperglycemia, hypertriglyceridemia and abdominal obesity. The mechanisms by which obesity increases sIgA concentrations are not known, but they are possibly associated with chronic low-grade inflammation, characterized by elevated concentrations of serum pro-inflammatory marker IL-6 [40]. IL-6 is one of the main cytokines in human milk, and its content has been shown to correlate with sIgA concentrations in colostrum in other studies [41, 42].

Unlike IgG, which is transferred transplacentally, the action of immunoprotective components of colostrum and milk is usually local, in the newborn’s intestinal mucosa [3]. sIgA is able to inhibit bacterial adhesion and neutralize virus infection in the intestinal mucosa, preventing tissue damage and loss of energy [43] through a non-inflammatory process called immune exclusion [44]. The IgG antibodies activate the complement system and granulocytes and induce cytokine production, which results in inflammation. sIgA can also act as opsonin, signaling the presence of antigens to phagocytes by binding to the surface of bacteria and facilitating aggregation. The opsonizing activity of sIgA is of great biological significance, and given that colostrum is the secretion containing the highest concentration of this antibody class, it provides a complete micro-environment where components found in both its soluble portion and cells act together [3, 44].

The increased serum C3 concentrations in overweight and obese women and serum C4 in the obese group was not accompanied by an increase in these concentrations in colostrum. It was previously reported that obese individuals exhibit higher concentrations of circulating C3 [45, 46] and C4 [47]. The complement system consists of proteins that interact to provide many of the effector functions of humoral immunity and inflammation [45]. C3 and C4, the central components of the complement pathway of the immune system, are synthesized by stimulation of pro-inflammatory cytokines [46]. The C3 and C4 proteins are mainly produced in the liver, but they can also be synthesized and expressed in other tissues such as the adipose [47]. It has been suggested that diagnosis of chronic low-grade inflammation, which characterizes obesity, is responsible for activation of the complement system, which, in turn, would cause the associated metabolic complications [48].

Obese mothers exhibited higher concentrations of serum CRP, but not in colostrum. CRP secretion by the liver is stimulated by several inflammatory cytokines, which are released in response to trauma, infection and inflammation, and this protein rapidly reduces the resolution of these conditions [49]. Another study found an association between serum CRP concentrations and prepregnancy BMI [50]. High CRP concentrations in the amniotic fluid of obese mothers expose the fetus to high amounts of inflammatory mediators, which may contribute to fetal programming, account for various complications during pregnancy and impact health condition in adulthood [51].

It should be considered that these data were evaluated in one period of collection and only one milk maturation stage that may be considered a limitation of this study. It is necessary to continue investigations focusing on other factors that may be involved during breastfeeding of the mothers with BMI alterations.

## Conclusions

The data obtained in the present study support the hypothesis that metabolic changes promoted by obesity can change the biochemical and immunological parameters of breast milk. Nevertheless, we did not observe any changes that could cast doubt on the protection that breastfeeding provides to newborns or that could reflect the inflammatory state observed in maternal serum, because the only immunological component that increased in serum the obese women was IgA, which is known to be a non-inflammatory antibody. However, the increased calorie and fat content and glucose concentrations detected in colostrum from obese women deserve further attention.
